# Effects of visual flow direction on signs and symptoms of cybersickness

**DOI:** 10.1371/journal.pone.0182790

**Published:** 2017-08-04

**Authors:** Alireza Mazloumi Gavgani, Deborah M. Hodgson, Eugene Nalivaiko

**Affiliations:** 1 School of Biomedical Sciences and Pharmacy, University of Newcastle, Newcastle, New South Wales, Australia; 2 School of Psychology, University of Newcastle, Newcastle, New South Wales, Australia; Tokai University, JAPAN

## Abstract

Our objective was to assess the influence of visual flow direction on physiological changes and symptoms elicited by cybersickness. Twelve healthy subjects (6 male and 6 female) were exposed to a 15-min virtual ride on a rollercoaster on two different days in a counterbalanced manner, such half of participants were facing forward during the first ride while another half was facing backward. Forehead skin conductance, heart rate and HRV parameters (SDRR, RMSSD) were collected as objective measures; subjective symptoms were assessed with the Motion Sickness Assessment Questioner immediately after exposure. We found that while nausea ratings at which participants terminated the experiment did not differ between forward/backward rides, the mean ride tolerance time was significantly longer during reverse ride compared to forward ride (6.1±0.4 vs 5.0±0.5 min, respectively, p = 0.01, η^2^ = 0.45). Analysis of HRV parameters revealed significant reduction in both RMSSD (p = 0.02, t = 2.62, η2 = 0.43) and SDRR (p = 0.01, t = 2.90, η2 = 0.45) in the forward ride; no such changes were found in the backward ride. We also found that amplitude of phasic changes in forehead skin conductance increased significantly in both ride directions. This increase however was significantly lower (p<0.05) in backward ride when compared to the forward ride. When assessed immediately post-ride, subjects reported significantly lower (p = 0.04) subjective symptom intensity after the reverse ride compared to the forward ride. We conclude that the direction of visual flow has a significant effect on the symptoms reported by the subjects and on the physiological changes during cybersickness.

## Introduction

Motion sickness (MS) develops when conflicting signals are received from the spatial orientation senses [1]. This could either be visual/vestibular/proprioceptive conflict such as when on a boat in a rough sea or can be initiated within a single sensory system such as canal-otolith interaction during Coriolis cross-coupling (rotation around vertical axis with head tilts) [1], or by purely visual stimuli such as optokinetic drum [2]. Apart from real motion, MS could be provoked by other means. Simulator sickness is frequently experienced by pilots who undergo simulator training [3, 4]. Cybersickness is a form of MS which is provoked by exposure to virtual reality. The principal symptoms of MS are well known and include cold sweating, facial pallor and nausea with potential vomiting [5]. Detailed previous studies however revealed that the list of MS symptoms is substantially longer. It is now accepted in the field that MS is a multidimensional syndrome, and that all its symptoms could be split into four clusters: gastrointestinal (stomach awareness, nausea, vomiting); central (fainting, light headiness, disorientation, dizziness, sensation of spinning); peripheral (sweating, feeling hot) and sopite (annoyance, drowsiness, tiredness, uneasiness) [5]. Individual’s susceptibility to MS varies greatly and depends both on the scale of provocation and individual factors such as sex, age and ethnic background [6, 7]. Additionally, there is now solid evidence suggesting that affective states such as anxiety may also contribute to MS susceptibility [8, 9].

Some studies have concluded that cybersickness induces more severe symptoms compared to simulator sickness [10]. Cobb *et al*. [11] discovered that 80% of subjects experiencing virtual reality felt some level of nausea in the first 10 min of the exposure. There are various technical aspects of virtual reality that can contribute to the induction of cybersickness; they include field of view [12], exposure duration [13], mismatched motion (lag) between anticipated movement of visual field and the actual movement displayed in VR device [14]. It appears however that the most critical factor contributing to the development of cybersickness is the content of virtual reality, in particular the amount of virtual motion. Not surprisingly, stationary visual scene was found to cause less symptoms compared to virtual oscillatory scene [15], and virtual double-axis rotation was more provocative compared to rotation around a single axis [16]. Another aspect of visual content that deserves attention is the direction of virtual movement. Very little is known on this subject, and we were able to identify just one publication that addressed it [17]. The authors presented to their subjects either expanding or contracting squares on a computer monitor, and found that expanding pattern (that elicited forward vection) caused more prominent MS and in larger number of subjects compared to contracting pattern (that elicited backward vection). Development of MS in this study cannot be explained in the framework of the classical “sensory conflict” theory as ideal constant linear motion would not elicit any sensations apart from visual, and thus there was no subject for a conflict. We thus aimed to test whether exposure to forward or backward virtual movement with multiple linear and angular accelerations, where vestibulo-visual conflict is apparent and inevitable, would have different effects. In our previous experiments we have established that out of several moving virtual environments, virtual ride on a rollercoaster is the most provocative one as only one of 14 volunteers managed to complete the ride while the rest terminated due to cybersickness [18].

Subjective rating of MS, like any psychometric score, lacks precision, and our secondary aim was to improve the accuracy of our study by adding objective physiological assessment. For this purpose, we monitored skin conductance level (SCL)–an index of sweating rate. In our previous experiments where we used identical provocation, we compared SCL changes recorded from finger and forehead, and found that finger location is not suitable for documenting nausea as responses in this area were initiated by highly-arousing onset of virtual ride, when nausea was still absent [18]. In contrast, rise in SCL on the forehead did not occur until subjects reported mild/moderate nausea, similar to findings reported during vestibular provocations [19]. Furthermore, dramatic increase in nausea-related SCL phasic events on the forehead (>500% for the amplitude and >1000% for the frequency) was in sharp contrast with very moderate or no effect on heart rate or respiratory rate [18]. These finding indicate that forehead SCL is the most sensitive and relatively specific biomarker of nausea (providing potential effects of intense physical exercise or overheating are excluded). In conclusion, the aim of this study was to investigate the effect of direction of visual flow on the intensity signs and symptoms of cybersickness, with complementing nausea subjective score by quantification of forehead SCL.

## Materials and methods

### Participants and experimental design

The study was conducted in 12 healthy volunteers aged 27±6 y.o., 6 females and 6 males. The study protocol was approved by the Human Research Ethics Committee of the Newcastle University. The participants were randomly assigned to two groups (n = 6 each); on two different days (at least one week apart) subjects experienced a virtual ride on a rollercoaster. The experiment was designed in a counterbalanced manner, so that participants from one group experiences forward ride on the first experimental day and backward ride on the second day, while the order was opposite in the second group.

On the day of arrival in the laboratory (air conditioned room kept at 21–22°C), after signing an informed consent, subjects completed the Motion Sickness Susceptibility Questionnaire, MSSQ [20]. After fitting head-mounted virtual reality display (Oculus Rift DK1, Oculus VR, USA), a 5-min baseline recording of heart rate and forehead skin conductance was performed. During this period, a stereoscopic neutral image was displayed. Subsequently, the forward or reverse video rollercoaster ride (Helix, Archivision, NL) was activated. The ride lasted for 15 min or until subjects felt too uncomfortable to continue, whichever came first. Subjects were asked to keep their heads as stable as possible during the ride. Subjects provided a verbal rating of nausea every minute, during the ride, on a scale from zero (no effect) to 10 (just about to vomit). After the ride, subjects completed the Motion Sickness Assessment Questionnaire, MSAQ [5].

### Data acquisition and analysis

ECG was measured from the lead II of a 3-lead electrode configuration. Forehead skin conductance level (SCL) was measured using 8-mm silver/silver chloride gel-filled self-adhesive electrodes connected to the constant voltage Model 2701 BioDerm Skin Conductance Meter (UFI, Morro Bay, USA). The electrodes were placed on the right and left sides of the forehead 1 cm bellow the hairline, near the lateral corners of the eyes. All sensors were connected to a PowerLab 8 data acquisition system and a computer running Chart 8.0 (ADInstruments, Sydney, Australia). Sampling rate was 100 Hz for skin conductance signals and 1 kHz for ECG. Heart rate and two cardiac vagal indices (SDRR and RMSSD) were computed from the ECG trace for each minute of recordings using HRV module of the Chart 8.0 software. To compute the phasic component of the skin conductance, we applied a high pass-filter with a cut-off frequency of 0.05Hz [19]. Chart software was also used to calculate the amplitude Root Mean Square (RMS) and frequency of the SCL phasic components.

Two types of analysis of physiological parameters were performed: i) dependence of measured variables on riding time; and ii) dependence of measured variables on nausea rating. As all participants terminated their ride at different times, we could not perform overall averaging of their data traces; instead, two points were selected for comparison: baseline (before the ride), and the last minute of the ride (i.e. when the nausea level was the highest). For the second type of analysis, data were split into “no nausea” (rating 0), “light nausea” (rating 1–3), “moderate nausea” (rating 4–6) and “strong nausea” (rating >6) bins.

Prism 7.1 (GraphPad, USA) was used for statistical analysis. Two-way ANOVA and T-tests for repeated measures were used to determine the effects of i) direction and time on physiological recordings and ii) nausea rating and direction on physiological recordings. T-tests for repeated measures were used to determine the effect of the ride direction on the slope of nausea rating vs. time relationship and effect of ride direction on ride duration period. The slope of nausea rise vs. time was determined by a linear fitting procedure according to the formula (Nausea rating = m x Time + c), where m is the slope; fitting procedure was performed based on all available data points from each subject. Data are presented as means ± standard error of the mean (SEM). Statistical significance was set at p<0.05.

## Results

### Effects of virtual ride on nausea levels

**Fig 1:**
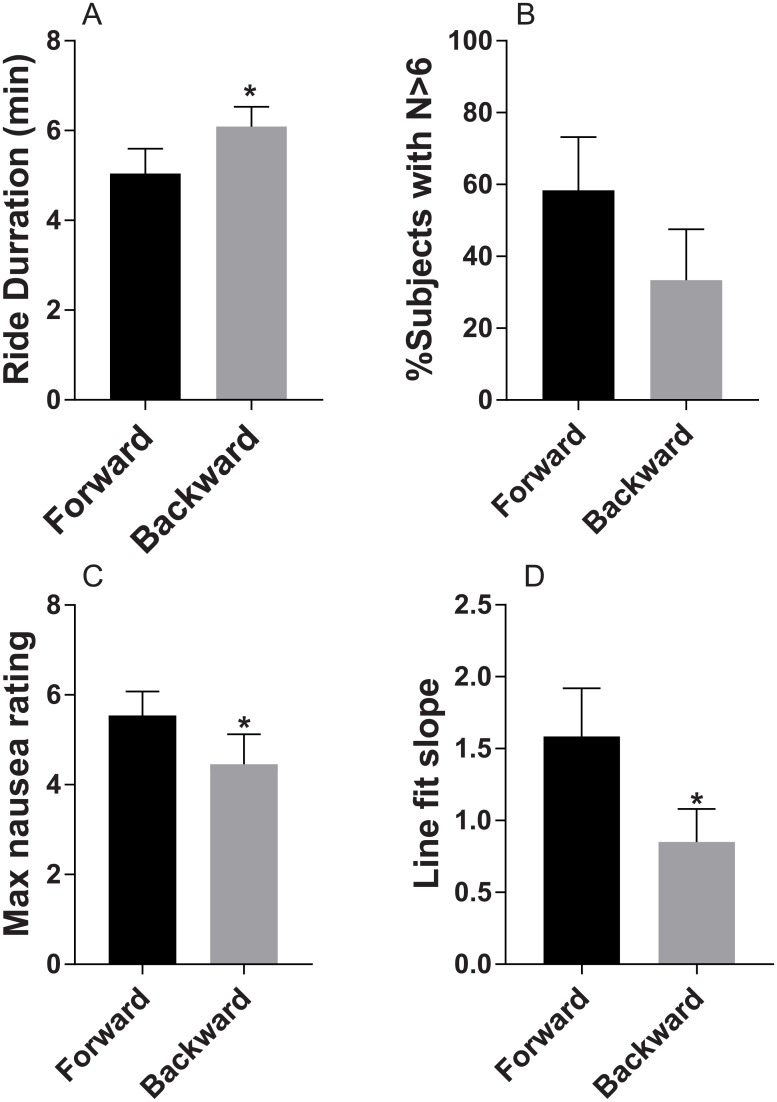
Reversing direction of virtual ride prolongs ride tolerance time and reduces real-time nausea scores. A—Average ride duration in forward and backward rides. B—Number of subjects reporting high level of nausea. C—Maximum nausea reported during the ride. D—Average slope of linear regression for individual’s nausea rating vs. time. *—P<0.05.

Participants differed substantially in their MSSQ score; it ranged from 11 to 36 (mean = 19.7 ± 12.0). All participants reported vection and some level of nausea during the ride. None of the participants managed to complete the 15-min ride in either directions; however the mean ride tolerance time was significantly longer during reverse ride compared to forward ride (6.1±0.4 vs 5.0±0.5 min, respectively, p = 0.01, η^2^ = 0.45, Fig 1A). Average nausea rating was zero before the ride in both experimental days. In the backward ride only 33% of the participants reported high levels of nausea while in the forward ride this increased to 58% Fig 1B. Although differences in average nausea ratings were not significant, there was a significantly lower maximal nausea rating when participants experienced reverse vs. forward ride (4.5± 0.6 and 5.5±0.5, respectively; p = 0.014, t = 2.96, η^2^ = 0.46, Fig 1C). There was also a significantly lower “nausea rating vs. time” slope during the reverse compared to forward ride (0.85±0.23 vs. 1.58±0.33 units/min, respectively, p = 0.04, t = 2.23, η2 = 0.33; Fig 1D).

### Subjective symptoms induced by virtual ride

**Fig 2:**
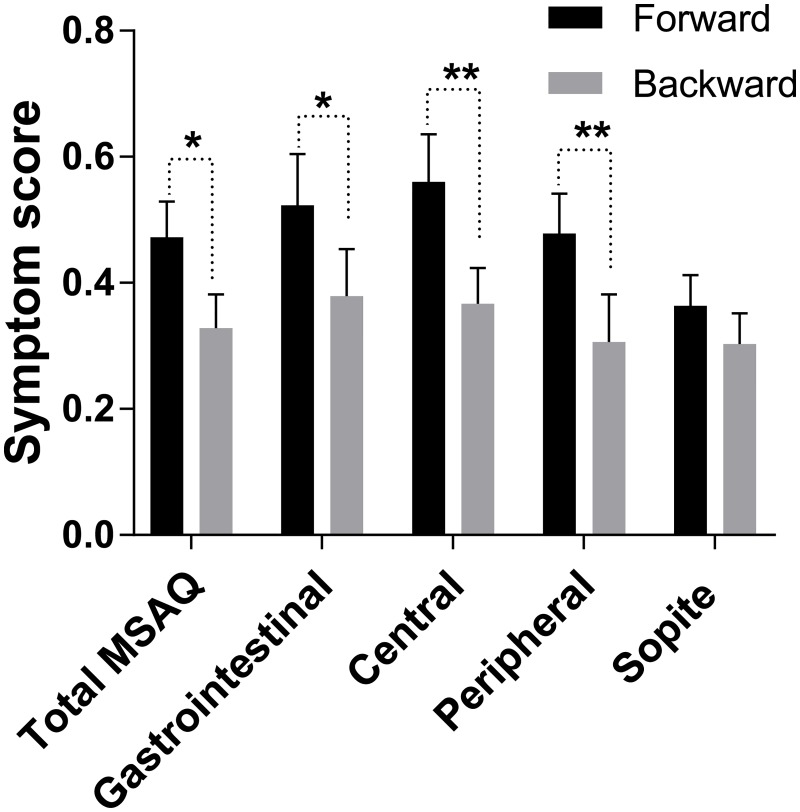
Subjective symptoms of cybersickness depend on the direction of virtual ride. From left to right total: MSAQ score, gastrointestinal symptoms, central symptoms, peripheral symptoms and sopite symptoms. * and **—p<0.05 and p<0.01, respectively.

When assessed immediately post-ride, subjects reported significantly lower symptoms intensity after the reverse ride when compared to the forward ride (Table 1 and Fig 2). Reverse ride direction was associated with significant reduction in total MSAQ score as well as with sub-scores in Gastrointestinal, Central and Peripheral symptom clusters. Sopite-like symptoms followed the same trend but the changes were not significant.

**Table 1:** Subjective symptoms of cybersickness depend on the direction of virtual ride.

	Forward (Mean±SEM)	Reverse (Mean±SEM)	Significance
Total MSAQ	0.47±0.05	0.32±0.05	p = 0.04, t = 2.75
Gastrointestinal	0.52±0.08	0.37±0.07	p = 0.04, t = 2.75
Central	0.56±0.07	0.36±0.05	p = 0.002, t = 3.7
Peripheral	0.47±0.06	0.30±0.07	p = 0.009, t = 3.2
Sopite	0.36±0.04	0.30±0.04	NS

### Effects of virtual ride time on physiological parameters

**Fig 3:**
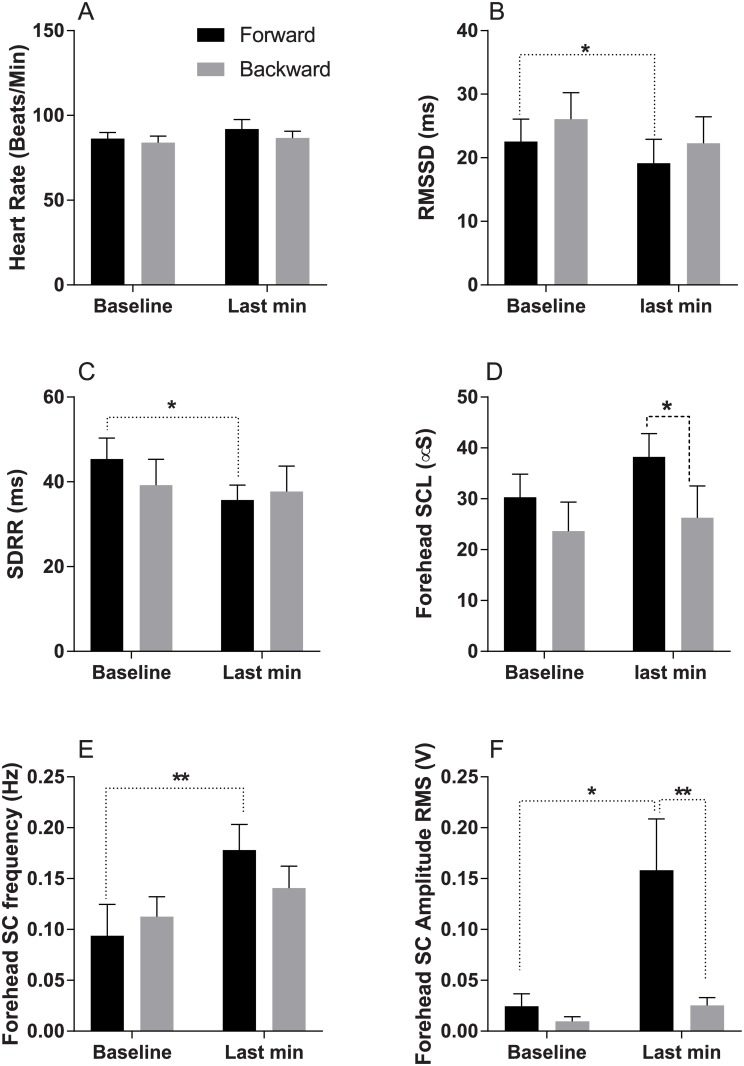
Changes in physiological parameters during forward (black bars) and backward (grey bars) virtual rides. Each graph shows data values for the last minute of baseline period and for the last minute of simulated ride. A—Heart rate; B—Root Mean Square of the Successive Differences in R-R intervals (RMSSD); C—Standard Deviation of RR interval (SDRR); D—Forehead tonic (DC) skin conductance; E—Forehead phasic skin conductance spike frequency; F—Forehead phasic (AC) skin conductance spike amplitude RMS. * and **—p<0.05 and p<0.01, respectively.

In this analysis pre-ride physiological parameters were compared to the values obtained during the last minute of the ride, when participants reported their highest nausea scores. There was no difference in baseline heart rate values recorded during forward and backward ride, and no effect of the provocative VR exposure oh heart rate (Fig 3A). There were also no differences in baseline RMSSD and SDRR values in the two conditions. There was however a significant reduction in both RMSSD (p = 0.02, t = 2.62, η2 = 0.43) and SDRR (p = 0.01, t = 2.90, η2 = 0.45) in the last minute of the ride compared to baseline in the forward ride; no such reduction was found in the backward ride (Fig 3B and 3C).

**Fig 4:**
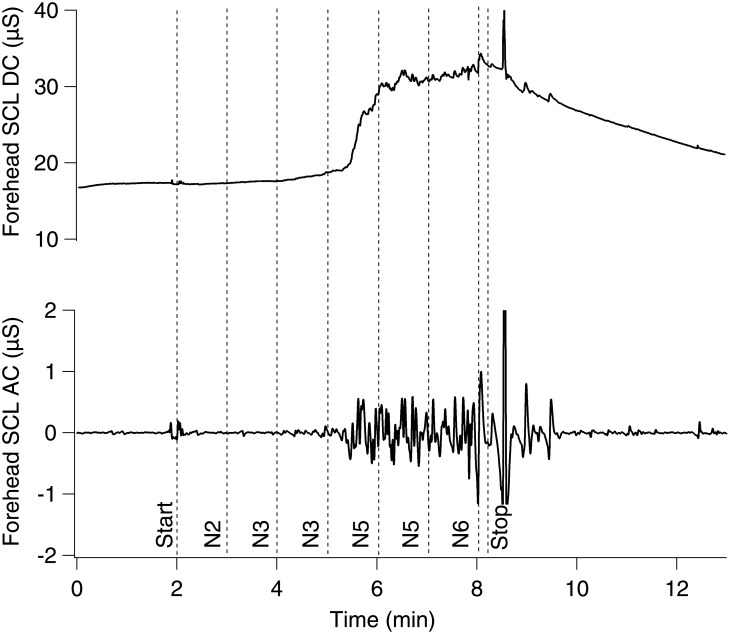
Example of forehead skin conductance recordings obtained in one subject during simulated ride. Top trace—tonic SCL; bottom trace—phasic SCL. Vertical lines depict start/stop of the virtual ride and nausea ratings; prior to the ride onset nausea rating was zero.

An example of the forehead SCL recordings obtained in one subject during a simulated ride is shown in Fig 4. There was no, or minimal (<1 event/min), skin conductance activity during baseline on either of the days. Forehead skin conductance phasic events gradually appeared during simulated ride; this occurred at different times, and their appearance was clearly associated with nausea development as illustrated in Fig 4. Frequency of spikes increased significantly (p = 0.01, t = 3.13, η2 = 0.49) only in the forward ride while this increase was not significant in the reverse direction (Fig 3E). Spike amplitude RMS value showed significant increase in the last minute of the ride when compared to the baseline in the forward (p = 0.03, t = 2.49, η2 = 0.38) and reverse (p = 0.02, t = 2.68, η2 = 0.39) directions, however, in the reverse ride spike amplitude RMS value was significantly (p = 0.01) less when compared to the forward ride, (Fig 3F).

### Effects of nausea level on physiological parameters

**Fig 5:**
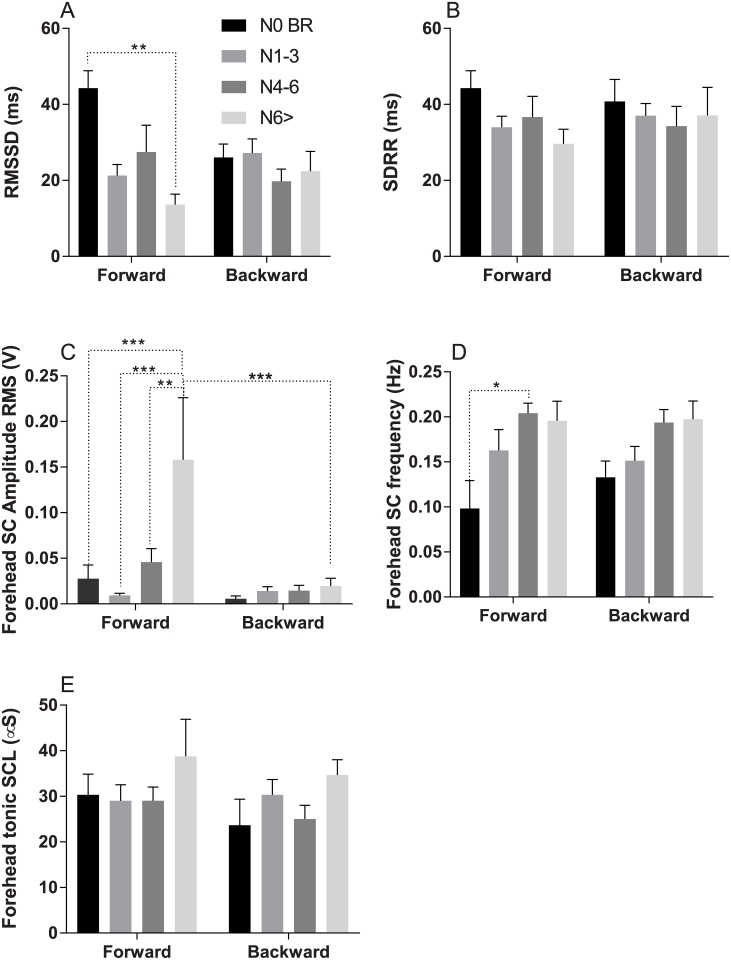
Dependence between nausea ratings and physiological measures. A—Root Mean Square of the Successive Differences (RMSSD) B—Standard Deviation of RR interval (SDRR) C—Forehead phasic (AC) skin conductance spike amplitude RMS, D—Forehead phasic skin conductance spike frequency. E—Forehead skin conductance tonic values. *, **, ***- p<0.05, p<0.01 and p<0.001 respectively.

RMSSD measure in the forward ride was inversely correlated with increasing nausea and reduced significantly (p = 0.001, F (3, 39) = 6.578) in high nausea (N>6) ratings, Fig 5A. There was no change in RMSSD measure in the reverse ride. SDRR changes did not show any correlation with nausea ratings in either of the ride directions, Fig 5B.

Forehead skin conductance spike amplitude RMS increased significantly (p = 0.001, F (3, 34) = 6.428) during nausea experience in the forward ride. This increase correlated with increasing nausea. The increase in spike amplitude was not significant in the reverse ride. The RMS spike amplitude decreased significantly (p< 0.001) in high nausea level (n>6) in the reverse ride compared to forward ride, Fig 5C. The frequency of forehead skin conductance events increased significantly (p = 0.015, F (3, 37) = 3.918) during nausea experience in the forward ride. This increase correlated with increasing nausea. This increase in spike frequency was not significant in the reverse ride, Fig 5D. Forehead tonic skin conductance events did not change in any of the ride directions, Fig 5E.

## Discussion

### Effects of visual flow on subjective symptoms

The aim of the current study was to determine the effect of direction of visual flow on autonomic changes and subjective symptoms during exposure to provocative virtual motion. Our measures included both subjective rating and objective biomarker of nausea—forehead skin conductance that closely correlates with nausea levels during both real motion-induced and VR-induced motion sickness [18, 19]. For assessing subjective symptoms, we utilized the well-established psychometric tool, MSAQ, that allows symptom classification in four different clusters [3, 21, 22]. In line with our previous work, where we used just a forward virtual ride as a provocative stimulus [18], we found that the predominant MS symptoms were the gastrointestinal ones, followed by the sopite, the central and the peripheral symptoms (see Methods for definitions). In our current study we employed the same MSAQ immediately after exposure to the roller coaster ride in both forward and reverse directions. We found that there was a significant increase in all clusters of symptoms and in the total MSAQ score in both directions when compared to baseline. These finding are in accordance with previous observations [3, 18, 21] that cybersickness can result in a multidimensional spectrum of symptoms. We also found that motion sickness severity was less after backward compared to forward virtual motion exposure. Total MSAQ score and all but one of the symptom clusters scores showed a significant reduction after the backward ride when compared to the forward ride. Tolerated ride duration was substantially prolonged during the reverse sessions, with reflecting slower time course of nausea development; this was paralleled by the gradual reduction of other symptoms as revealed by MSAQ scores.

### Effects of visual flow on autonomic parameters

We found that heart rate did not change with increasing nausea; this finding is in line with most previous studies [18, 19, 23] that reported minimum or no changes. We also assessed two HRV parameters, RMSSD and SDRR; both reflect vagally-mediated components of HRV and are associated with short-term changes in heart rate. Time-domain HRV parameters are generally calculated for a minimum of 5-min interval [24] however there are many studies confirming that values obtained from a shorter (1-min) intervals correlate well with the results calculated from a standard 5-min recording [25–28]. Since the ride time in our experiments was dependent on the subject and many of the volunteers tolerated only few minutes of the ride, and also since the symptoms progressed rapidly, a 5-min recording was not suitable for this study, and we used a 1-min epochs for computing HRV parameter. We found that RMSSD and SDRR decreased significantly during forward ride and remained unchanged during the reverse ride, suggesting that forward ride was associated with the suppression of cardiac vagal tone.

Previous studies, including our own, reported that phasic skin conductance activity in the forehead area is a reliable way of quantifying levels of nausea [18, 19]. There was no or minimum phasic activity during baseline recording (at zero nausea ratings), and in majority of the subjects phasic activity started or augmented with increasing nausea. One of major findings in this study was that there was a significant increase in forehead spike amplitude and frequency in the last minute of the forward ride, when nausea ratings were maximal. Although average RMS signal increased in the reverse ride, this rise was not significant. Furthermore, changes in frequency of the phasic events were significantly higher in the forward direction of the ride when compared to the backward ride. These findings paralleled subjective ratings of nausea, which show higher nausea ratings in the last minute of the forward ride when compared to the reverse ride.

Our results thus represent the first report presenting evidence that moving forward in a motion-rich virtual context appears to be more provocative than moving backwards; this is reflected in both subjective and objective signs of cybersickness. In the following sections we present our view on the potential causes underlying this difference.

### Ride direction as a provocative factor

Susceptibility to cybersickness varied substantially between our subjects as determined both by subjective and objective measures. As none of our participants had any previous experience with VR, these differences were clearly unrelated to previous exposures. It is not excluded that differences in symptoms were in part due to potential differences in head movements during the ride. Lack of assessing this parameter is a study limitation; it is however unlikely that the amount of head movements contributed to sensory conflict, due to the very nature of the VR hardware and software that matched voluntary induced shifts in the virtual visual field due to head rotations and tilts, thus preventing vestibulo-visual sensory mismatch. The true vestibulo-visual conflict was between virtual linear and angular accelerations and lack of corresponding sensations from the vestibular receptors.

There are various features of VR technology that could be responsible for inducing cybersickness; generally these factors can be classified into three classes: hardware-dependent (lack of head position tracking and visual field movenet in XYZ planes, a lag between head move and visual field move, monitor flicker, disaccord between vergence and accommodation), content-dependent [29], and mismatch caused by subject movements. In our case, the dominant contribution of the content (roller coaster ride) is evident from the fact that observing the static image during baseline period did not provoke any discomfort. Throughout the experiment in forward and backward ride subjects were seated on a stationary office chair with limited movement capability, and thus their movement was limited to head movements or in some cases upper body movements. Since the main technical aspects (field of view, display resolution) of both forward and backward ride directions were identical, we conclude that the major factor responsible for differences in the “provocativeness” between the two conditions was the direction of visual flow.

### Why forward ride is more provocative?

A possible explanation for the finding that forward virtual ride is more provocative than backward is that here there may be some parallels with the dependence of motion sickness on the ride direction in land vehicles in the real world. While we were not able to identify any research publication on this subject, it is common knowledge that sitting backward to the direction of travel often causes nausea; in fact it is not uncommon to find is social media (e.g. Trip Advisor) requests to advise how to book forward-oriented seats on trains. Whatever the mechanism of motion sickness in this latter case, it must be different from the one observed in the current study because sitting backwards in the virtual world appeared to be less, not more provocative.

It is quite probable that the core mechanism responsible for the phenomenon described in the current study may include some higher neural functions including complex cortical analysis of moving visual scene. Several previous studies explored effects of expanding and contracting geometric patterns that provoke sensations of forward and backward self-motion (vection), respectively. It appeared that the threshold for vection was lower for contracting patterns [17, 30], and providing vection is positively correlated to motion sickness [10], one would expect that a contracting pattern (simulating backward motion) would be more provocative. In contrast to this reasoning, but in accordance with our results, Bubka *et al*. [17] reported that an expanding pattern was in fact more provocative. Precise neural mechanisms responsible for these differences are yet to be identified. Of note, results of described experiments do not fit into the framework of sensory conflict theory as linear motion at constant velocity does not elicit any vestibular activation and thus there is no condition for sensory mismatch.

An alternative explanation for the differences in the provocative effect of visual flow associated with its direction could be that that during a forward-facing ride, anticipation of vigorous falls and turns that could be seen and anticipated in advance provoked hyperarousal locked with an anxiety- or fear-like state. The latter facilitated development of motion sickness induced by mismatch between intense visual flow and lack of vestibular sensations. Indeed, close association between anxiety and nausea is well documented: Tucker and Reinhardt [31] showed higher state anxiety in airsick students during flight training compared to non-airsick, and Collins and Lentz [32] reported a higher trait anxiety in motion sickness susceptible subjects. In a large community survey conducted by Haug *et al*. [33], among other risk factors, anxiety had the highest predictive power for nausea. This link between anxiety and nausea appears to be bidirectional as Eagger *et al*. found that patients with vestibular disease often report anxiety symptoms [34]. In our previous study with identical virtual ride on a rollercoaster we found that ride onset was associated with moderate tachycardia and tachypnea reflecting arousal. Reduction of cardiac vagal HRV indices reported here during forward but not during backward ride supports the idea that the former condition was associate with anxiety state while the latter was not. There is rich literature linking reduced vagal tone to anxiety, and general consensus is that in subjects with affective disorders such as post-traumatic stress disorder, generalized anxiety and other anxiety disorders, time-domain vagal indices are reduced [35, 36]. A meta-analysis on 36 relevant studies concluded that there is an overall reduction in HRV parameters in patients with various forms of anxiety disorders [37]. It must be however acknowledged that cardiac vagal activity is also reduced by non-affective provocative visual stimuli [38].

### Conclusions and perspectives

Our findings clearly demonstrate that the direction of visual flow in virtual stimulation has a significant effect on the symptoms reported by the subjects and the physiological changes during cybersickness. We propose a hypothesis that the underlying mechanism responsible for the more provocative potential of the forward direction may involve induction of an anxiety-like state that potentiates effects of sensory mismatch. It would thus be of major interest to test in future experiments whether there is an association between state anxiety and sensitivity to virtual motion.
